# Development of a brief multidisciplinary education programme for patients with osteoarthritis

**DOI:** 10.1186/1471-2474-12-257

**Published:** 2011-11-11

**Authors:** Rikke H Moe, Espen A Haavardsholm, Margreth Grotle, Eldri Steen, Ingvild Kjeken, Kåre Birger Hagen, Till Uhlig

**Affiliations:** 1 National Resource Centre for Rehabilitation in Rheumatology, Diakonhjemmet Hospital, PO box 23 Vinderen, 0319 Oslo, Norway; 2 Department of Rheumatology, Diakonhjemmet Hospital, PO box 23 Vinderen, 0319 Oslo, Norway; 3 Institute of Health and Society, Faculty of Medicine, the University of Oslo, Norway; 4 FORMI, Clinic for surgery and neurology (C1), Oslo University hospital, Ullevaal, 0407 Oslo, Norway

## Abstract

**Background:**

Osteoarthritis (OA) is a prevalent progressive musculoskeletal disorder, leading to pain and disability. Patient information and education are considered core elements in treatment guidelines for OA; however, there is to our knowledge no evidence-based recommendation on the best approach, content or length on educational programmes in OA. Objective: to develop a brief, patient oriented disease specific multidisciplinary education programme (MEP) to enhance self-management in patients with OA.

**Method:**

Twelve persons (80% female mean age 59 years) diagnosed with hand, hip or knee OA participated in focus group interviews. In the first focus group, six participants were interviewed about their educational needs, attitudes and expectations for the MEP. The interviews were transcribed verbatim and thereafter condensed.

Based on results from focus group interviews, current research evidence, clinical knowledge and patients' experience, a multidisciplinary OA team (dietist, nurse, occupational therapist, pharmacist, physical therapist and rheumatologist) and a patient representative developed a pilot-MEP after having attended a work-shop in health pedagogics. Finally, the pilot-MEP was evaluated by a second focus group consisting of four members from the first focus group and six other experienced patients, before final adjustments were made.

**Results:**

The focus group interviews revealed four important themes: what is OA, treatment options, barriers and coping strategies in performing daily activities, and how to live with osteoarthritis. Identified gaps between patient expectations and experience with the pilot-programme were discussed and adapted into a final MEP. The final MEP was developed as a 3.5 hour educational programme provided in groups of 6-9 patients. All members from the multidisciplinary team are involved in the education programme, including a facilitator who during the provision of the programme ensures that the individual questions are addressed. As part of an ongoing process, a patient representative regularly attends the MEP and gives feedback concerning content and perceived value.

**Conclusion:**

A MEP has been developed to enhance self-management in patients with OA attending a multidisciplinary OA outpatient clinic. The effectiveness of the MEP followed by individual consultations with members of the multidisciplinary team is currently evaluated in a randomised controlled trial with respect to patient satisfaction and functioning.

## Background

Osteoarthritis (OA) is by far the most prevalent joint disorder and is associated with pain, functional disability, and impaired quality of life. In the Version 1 estimates for the Global Burden of Disease 2000 study, published in the World Health Report 2001, osteoarthritis is the 6^th ^leading cause of years lost to disability at a global level and accounting for 3.0% [1]. The prevalence is higher among women than men and increases with age [2,3]. Several factors contribute to the risk of osteoarthritis, including age, gender, genetics, behavioural influences, obesity, injury and reduced muscular strength [4]. Significant consequences of OA are activity limitations, reduced participation in work and social activities, and mental distress. The exact incidence is difficult to determine and varies dependent on the population studied and the diagnostic methods in use [3].

Available treatments for OA include pharmacological, non-pharmacological and surgical care, and are mainly aimed at alleviating symptoms and functional consequences. At present no disease-modifying interventions are available [5]. Guidelines for treating OA recommend a combination of pharmacological and non-pharmacological treatments [6,7]. Patient information and education are considered core treatments for OA in evidence based clinical guidelines [8], but no agreement exists on the content, description and implementation of educational programmes. One obvious reason for the lack of agreement might be that there are few studies exploring which elements in patient education programmes that contribute to a documented effect in trials. Another reason is the lack of detailed guidance on how to describe the content of interventions in research studies; this is in particular a challenging task in multidisciplinary interventions and non-pharmacological interventions [9,10].

Many different patient education programmes have been developed for health conditions including arthritis, and these may be using a disease specific or generic approach, some are group-based and some are individual, and they may be given by health professionals or lay tutors [11]. In addition to increased knowledge and patient empowerment, these programmes also aim at changing health behaviour, by teaching patients how to solve problems and set individual goals [12]. Reviews indicate that patient education programmes can improve knowledge, change behaviour and improve health outcomes in some chronic conditions, but the magnitude of effect varies [13-15]. The evidence of effect of patient education programmes for OA in general is inconclusive [12,16,17], and the overall effect size for improving pain and functioning on a short term basis is at best small (0.06, 95%CI 0.02-0.10) [18]. Even if short-term effects in some programmes are observed, long-term changes in health status are not convincingly demonstrated. In addition to the effects on health status, it has also been shown that patient education and self-management programmes for knee osteoarthritis can improve psychological outcomes [19].

The most widely known example of patient educational programmes for arthritis is the Chronic Diseases and Arthritis Self-Management programmes (ASMP) developed at Stanford University in the United States. The ASMP has been generally tested with positive results [12] but a randomized controlled trial investigating the effectiveness of the programme for patients with arthritis did not demonstrate any significant benefits at four months follow-up [20]. ASMP are given in groups, often led by lay tutors, they have a generic approach and the groups are not disease specific [11]. Up until 2005 ASMP and similar programmes contributed to the main body of educational programmes in OA. Other patient education programmes combine exercise and patient education [21], include the spouse with the aim of increasing coping skills [22], describe mail delivered leaflets [23] or individual telephone-based [24] programmes. The group size and description of the educational content is often not reported in detail, and the length of the programmes varies.

Core concepts of patient self-management have been described as engagement in activities which promote health; monitoring physical and emotional status, appropriate interaction with healthcare providers; and management of the effects of illness on emotions, self-esteem and relationships with others [25,26]. Patients and health professionals may view patients' needs differently [27]. In summary, current patient education programmes in general vary in form and content, but the majority of these interventions are led by health professionals in a group setting where most participants are affected by a similar condition, and all components of the intervention can be tailored to specific needs of the group. There is to our knowledge no evidence that longer programmes with extended meeting points are more effective or feasible than brief patient education programmes.

The objective of this study was to develop and describe a brief, patient oriented disease specific multidisciplinary education programme (MEP) which could be used to enhance self-management in patients with moderate to severe OA.

## Methods

### Searching for evidence-based information

This study included evidence-based advice and information with the highest levels of evidence available. The literature was reviewed for systematic overviews for non-pharmacological and non-surgical treatment strategies for hand [28], hip [29] and knee OA [19], recommendations for the management of osteoarthritis [7,8,30] and systematic overviews on the effect of different patient education programmes [11,31]. In addition to this, a systematic search was performed to collect information on existing group-based educational programmes in OA.

### Focus group interview

Twelve patients (80% females, mean age 59 years) who had been treated by a multidisciplinary team at an osteoarthritis outpatient clinic within the past 12 months participated in two focus group interviews. The patients were recruited using critical case sampling, selecting active, critical and engaged patients believed to yield most variable information and depth. The multidisciplinary OA team suggested inclusion to the focus-groups, and the qualitative researcher invited the patients to participate. The focus groups were led by a researcher with experience in qualitative research (ES) and aimed at exploring the participants' a) educational needs, b) attitudes and c) their expectations for the MEP. The qualitative researcher was not involved in the patient intervention and did not have a dual role during the interviews. All patients involved in the project signed an informed consent and were informed according to the Helsinki declaration. The data inspectorate and the regional ethical committee (REK) approved the project (REK ref 156-06073 1.2006.598).

In the first focus group, six participants were interviewed by a researcher (ES) and an assistant about their educational needs, attitudes and expectations for the MEP. The qualitative approach applied was minimally structured open ended focus group interviews, theoretically framed within the tradition of phenomenology [32]. The interviews were recorded, transcribed verbatim and thereafter condensed (ES). The analytical strategy followed the six analytical propositions using categorisation, saturation and creating subcategories to explore the opinions and attitudes of the groups [33].

### Developing the content

Based on results from the current research evidence, focus group interviews, and clinical experience, the multidisciplinary OA team at a national centre for rehabilitation in rheumatology (dietist, nurse, occupational therapist, pharmacist, physical therapist, and rheumatologist) and a patient representative developed the content of a brief pilot-MEP. Based on the data from the first focus group, four main themes with thematically similar questions were identified and served as a guide for this process. For this purpose all members of the multidisciplinary team attended a two-day workshop in health pedagogics. The aim of the workshop was learning to use different teaching methods that could promote activity and participation in patients related to the topics in the MEP in a brief setting.

### The second focus-group

The pilot-MEP was tested in and evaluated by a patient group consisting of four members from the first focus group and six experienced patients. The focus group interview was performed immediately after provision of the programme. After the second focus group we concluded that there was sufficient variation in themes. Additionally, many of the themes from the first group also came up in the second group, indicating saturation of topics.

### Adjustments

**Figure 1:**
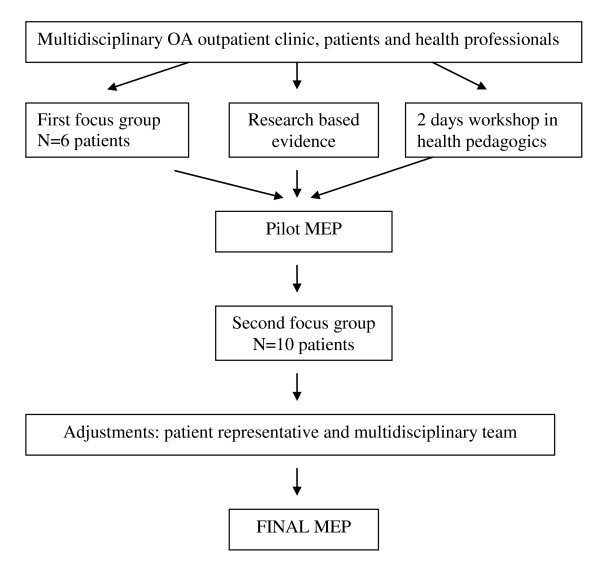
**Process developing the MEP**.

Data from the second focus group was transcribed and condensed (ES) then discussed with the multidisciplinary team and used for adapting the final MEP (Figure 1).

## Results

### Initial focus group interviews

The first focus group suggested that the most important themes for the patient education programme were as follow: a) What is OA, including knowledge about the disease processes and how OA is related to lifestyle and overweight b) Activity possibilities or limitations, knowledge about the symptoms related to the condition, and what the patients can do themselves to influence these, including suggestions for physical activity and exercise c) Treatment options including pharmacological, non-pharmacological, surgical and alternative treatments and their effects and possible interaction effects d) How to live with the condition to increase control, the possibilities of diets, advice on when and where to get help with the different symptoms and functioning problems in daily life. They also discussed experiences with the health care systems and underlined the need for a multidisciplinary approach to meet the individual challenges. One participant said "I was met as an individual and my problems were 'seen' when I was examined by a pharmacist and a dietist in addition to the rheumatologist".

A review of the literature of patient education and arthritis/osteoarthritis was performed including publications as by ultimo 2005. Findings from the literature review were discussed in the group and included in the health pedagogics workshop.

### Development and evaluation of a pilot MEP

Based on the identified themes and the compiled research evidence, a 3.5 hour pilot MEP was developed and implemented in a group of 10 patients. To ensure feasibility in a clinical setting the MEP was designed to be brief, stretching over a time period of no more than 4 hours, followed by individual multidisciplinary consultations in the outpatient clinic on the same day. The second focus-group took place immediately after the pilot-MEP. This resulted in some important identified gaps between patient expectations and their experience with and opinion about the pilot-programme. Patients expressed a wish for more practical information to enhance their own self-efficacy through more knowledge on different therapies and "Where to get help with different aspects of the disease" and "Navigating in the jungle of therapies" rather than discussing psychological consequences of living with a chronic disease. They also expressed a need for more focus on side effects and interaction effects of pharmacological treatments. The group participants also suggested diminishing the focus on how the disease affects relations, family and friends. One informant said: "We don't get past the surface of these kinds of problems in a short amount of time".

The possibility for more time to share between-patient experiences was also brought up. One informant reflected on this discussion in the focus-group interview: "Those of us who have been ill for a long time may have had a dream of passing on our own experiences." This resulted in an integrated lunch break without health professionals present, with the possibilities of dialogue with respect to sharing personal experiences without interference.

The identified gaps were discussed and through consensus integrated into the final MEP. One exception was however the gap between how much time three of the participants wished to allocate for the MEP (at least two full days) on one hand and attributable institutional resources on the other hand, which was also formulated in the aim of this study and in the initial mandate of the first focus-group.

All patients are seen individually after the group-based programme to address personal questions and examine them according to their needs.

### Modification of the MEP

The content and the practical application of the MEP were further evaluated in team group evaluations two times yearly. Adjustments were made to avoid individual patients becoming too dominant and too private in the group setting. A group meeting with the multidisciplinary team, the patient representative and the researchers concluded that the frames and expectations should be addressed in a more specific way. Thus, during the welcome address of the group it was underlined that all issues raised within the group based sessions should be relevant to others and that private issues should be noted in writing and addressed during the individual sessions later (Table 1 and 2). This small change in setting priorities for the content of the group session largely improved the working climate within the programme sessions.

**Table 1:** The OA educational programme

"Aim of the day": All attending patients should get an opportunity to update and optimize their knowledge about osteoarthritis (OA)	
13.00 H	**Welcome** Setting, norms and frames for the MEP Self-awareness exercise to collect patient expectations and wishes

13.20 H	**"Up to date" facts about OA** Education about the disease and disease processes, diagnosis, prevalence and causes. Overview of recommended treatments (Rheumatologist)

13.45 H	**OA medication** Pharmacological and herbal treatments, effects, possible interaction effects, side effects and level of evidence (Pharmacist)

14.10 H	**Tea break** Sharing experiences, reflections and discussion in the group

14.40 H	**Living with OA** What can one do to control symptoms in daily life? Treatment options including effects of non-pharmacological, surgical and alternative treatments (e.g. exercise, weight reduction, orthoses, devices) and when the different treatments are needed. Overview on how to get in touch with persons and systems who can provide help with these issues when applicable (Occupational Therapist & Physical Therapist)

15.30 H	**Break** Move your body

15.45 H	**OA Diet** The effect of different diets, supplements and weight reduction (Dietist)

16.10 - 16.30 H	**Discussion** Group questions for Rheumatologist, Pharmacist, Physical Therapist, Occupational Therapist and Dietist

16.30 -	**Outpatient clinic** All patients are seen individually by a rheumatologist and other health professionals dependent on identified needs.

**Table 2:** Tasks for the facilitator

Original tasks for the facilitator during the educational programme
Welcome, go through the programme, frames and expectations

Collect questions formulated by patients prior to the MEP

Engage in dialogue with "quiet" participants during the breaks

Seek answers to questions that are not being answered immediately in the group

### The final patient education programme

The final MEP is a 3.5 hour OA specific educational programme provided in small groups of six to nine patients. It addresses facts on OA, evidence based information on treatment options, strategies for pain management, recommendations for exercise and physical activity, general information on a healthy lifestyle, coping skills and options, and consequences of living with a chronic disease (Table 1). All members of the multidisciplinary team are involved in the programme, and a facilitator (Table 2) is present with the group during the whole programme to ensure that the questions formulated individually by each patient before attending the MEP are addressed by the different health professionals. One core element in the MEP is the "pedagogical sun" [34], where all patients write down their most important questions related to their OA, then rate the importance of each question, and finally share the top three issues with the group. Using the 'pedagogical sun' helps the health professionals in tailoring the MEP to the participants. Examples of what patients write down as their most important questions for the day are; "Should I go on a diet?", "How physically active can I be?" and "Can something be done to improve my tiredness?".

### Ongoing evaluation

Once every 6 months a patient representative attends a MEP to evaluate the content and methodology. Relevant issues which apply to the agreed intentions of the programme are discussed with the project leaders, and a short version is presented to the multidisciplinary team. Systematic searches on evidence based knowledge of OA and its treatments are also performed every year to update this part of the MEP.

### The multidisciplinary OA outpatient clinic

All patients are in conjunction with the group-based MEP seen individually by a rheumatologist and also, dependent on identified needs, by members of the multidisciplinary team.

## Discussion

We describe the development of a brief educational programme provided to patients with OA by a multidisciplinary team. The programme has specifically been developed to enhance self-management in OA. It was developed by experienced health professionals in cooperation with patient representatives to strengthen the patient perspective and ensure feasibility. The multidisciplinary team was convinced that to be effective, such interventions should be as acceptable to patients as possible. The basic knowledge of teaching methods and techniques from a workshop in health pedagogics was applied to the team to ensure active participation in a setting with patients.

Although the CONSORT statement underlines that the description of interventions should include" sufficient details to allow replication" [35], most complex interventions such as self management programmes are often insufficiently described to be replicated [36]. This hampers the possibility of comparing different programmes and results from trials involving patient education. The format of scientific journals as a rule does not allow providing a thorough description of the process and the content of the programme such as the MEP, which is necessary to enable clinicians and other researchers to implement an intervention.

Patient education is considered an integral part of self management programmes. It is assumed that patients use the acquired information for making changes in their lives and when taking decisions regarding treatment. Therefore it is important to know which information patients consider essential and necessary. Additionally, this study found that patients should be able to articulate their preferences, needs and expectations in order to translate the new information to their own context and environments. They also indicated a need for an overview of treatment options and information on how they could control symptoms in addition to knowledge about self management i.e. activity possibilities or restrictions and advice on how to live with the condition. Topics like improving the patient-doctor communication, availability of resources in health care and pain and fatigue management have also been reported to be of particular interest by patients in other studies [37]. Needs of patients with musculoskeletal diseases has previously been assessed and show comparable results as the results from the focus groups in this study. About 90% of the patients with OA, rheumatoid arthritis, back disease, lupus and systemic sclerosis wanted to learn about disease specific topics like various treatments, the illness, and what to expect from it [37]. The patients rated support from friends and family, their physician and exercise to be the most helpful factors in how to handle the diseases. Another study showed that receiving conflicting information regarding medications can result in increased concerns by patients and poorer adherence to recommended treatment in the future [38]. An advantage with an organised group intervention like this MEP is that patients receive the coordinated information and advice from all team members, avoiding redundant and conflicting information.

Integrating evidence based findings, clinical experience and the information needs formulated by the focus groups posed the challenge of integrating all contributions to one final programme. When in doubt, information gathered from patients' experiences in the focus groups was considered most important in order to ensure a patient oriented MEP. When addressing what OA is and providing an overview on treatment possibilities, however, the results from the literature searches, guidelines and the overviews served as a guide for this part of the MEP content. Evidence from research in the areas of hand, hip, knee and generalized OA were integrated. Much of the basic knowledge on OA is common for these locations, but where the evidence for treatment effects differs between the locations, the multidisciplinary team informs the patients about such differences.

This educational programme includes only one group session and is shorter than other published educational programmes for osteoarthritis (Additional File 1)[39-54], such as the ASMP, which implies meeting for six sessions or the Swedish programme [48] which implies meeting for 5 sessions. In comparison, the telephone based education programme by Allen et al [24] is given on an individual basis and implies 12 phone calls. Furthermore, the ASMP is generic in its form and often provided by lay tutors, whereas the MEP is disease specific and led by a multidisciplinary team of health professionals comparable to the Swedish version where the educational programme is given by different health professionals. The MEP is developed by patients and a multidisciplinary team of health professionals while the Swedish programme is developed by physical therapists and occupational therapists. It is possible that an extended MEP could have been more useful to patients; however a priority was to keep it focused, brief and feasible. This avoided conflicting and overlapping information. Keeping the programme as a total within the time limits of one day also made it more feasible for all patients to attend the programme, including the ones who work. Given that only 3.5 hours was used for the MEP, it was important that the time was used as efficiently as possible. To achieve this, groups were kept small to allow immediate interaction during the programme, presentations were short and included discussion themes, small breaks were introduced and the content tailored to the participants' formulated needs.

A weakness of the development process is that it was performed at one hospital centre only, and in a selected group of patients. Possibly patients from different geographical regions would have contributed differently to this process. The MEP should therefore be tested in other clinical settings. Secondly, in any interview situation there is a risk of "eager to please" bias. To reduce such a bias the interviewer in the focus groups was not involved in the patient care or in the development of the MEP contents. Also the interviewer directed questions on the different themes to the less expressive participants during the interviews to compensate for increased weight of more dominant focus group members. Involving only the active and critical patients could also have influenced the results, overestimating a need for detailed information.

The effectiveness of this brief educational programme has yet not been evaluated in a randomised, controlled trial. However, the MEP is currently evaluated as part of a multidisciplinary osteoarthritis outpatient approach in a large randomised controlled trial, in which it is compared to usual outpatient care, with respect to patient satisfaction, and functioning [55].

## Conclusion

A brief multidisciplinary educational programme for patients with OA has been developed, integrating knowledge from patient representatives, research and a multidisciplinary OA team.

## Competing interests

The authors declare that they have no competing interests.

## Authors' contributions

All authors were involved in planning and designing the study and ES was responsible for the qualitative methods used. ES, RHM, EAH and TU analyzed the data; all authors were involved in drafting and revising the manuscript and approved the final manuscript.

## Pre-publication history

The pre-publication history for this paper can be accessed here:

http://www.biomedcentral.com/1471-2474/12/257/prepub

## Supplementary Material

Additional file 1 **Appendix 1**. Group-based educational programmes for osteoarthritis in the literature. Click here for file
