# Supplementary material for: Development of a brief multidisciplinary education programme for patients with osteoarthritis
Source: BMC Musculoskelet Disord. 2011 Nov 11;12:257. doi: 10.1186/1471-2474-12-257 (PMC3262862; doi:10.1186/1471-2474-12-257)
Supplement: Additional file 1 — Appendix 1. Group-based educational programmes for osteoarthritis in the literature. [file 1471-2474-12-257-S1.DOCX]

Appendix 1: Group-based educational programmes for osteoarthritis in the literature*

| Author, year | Diagnosis | Group size | Duration of programme (sessions, hours) | Providers | Educational content |
| --- | --- | --- | --- | --- | --- |
| Arthritis Self Managment programmes (ASMP) | | | | | |
| Lorig (39-41) | Different diagnoses including OA | 8-20 participants | 6 x 2-2.5 h | Mostly lay tutors | Pain managment, exercise, diet, sleep, problem solving, medication, communication skills |
| Barlow, 1998 (42);  Barlow, 2000 (43) | Arthritis, including OA | NR | 6 x 2 h | Lay tutors | Version of ASMP for United Kingdom |
| Patel, 2009 (44); Buszewics, 2006 (45) | Hip and knee OA | NR | 6 x 2.5 h  + booklet | Lay tutors | Pain and fatigue, coping with feelings of depression, relaxation techniques, exercise, diet, communication skills, planning for the future |
| Cronan, 1997 (46) | Arthritis, OA symptoms | NR | 10 x 2 h  +  2h monthly x 10 | Professional health educators | ASMP + Information on when to see a health care professional, different remedies |
| Other programmes | | | | | |
| Maurer,1999 (47) | Knee OA | NR | 4 sessions | Health professionals | Disease process and clinical characteristics, video about joint protection, diet, guide to community services and  coping with pain and disability |
| Ekvall Hansson, 2010 (48) | Knee, hip or hand OA | NR | 5 x 3 h | Health professionals | Anatomy, symptoms, how to control the symptoms, ergonomics, diet, physical activity, exercise |
| Coleman, 2008  (12;49) | Knee OA | 8-10 patients | 6 x 2.5 h | Nurse | Disease information, pain managment, exercise, balance, joint protection, diet, medication, coping, SMART goals |
| Victor, 2005 (50) | Knee OA | 6-8 patients | 4 x 1 h  + home visit | Nurse | Disease information, treatments, exercise, relaxation, coping |
| Keefe, 1999 (22) | Knee OA | NR | 10 x 2 h | Health professionals | Cognitive and behavioural  pain coping strategies.  With or without spouse |
| Lord, 1999 (51) | Knee OA | Max 6 patients | 4 x 4 h  + individual pre- and post- programme home visits | Nurse | Disease information, pain relief, diet, joint protection, exercise, relaxation, problem solving |
| Fernandes, 2010 (52) | Hip OA | 6-8 patients | 3 x 1.5 h  + individual sessions pre and post –group | PT | Disease information, treatments, exercise |
| Calfas, 1992 (53) | OA | NR | 10 sessions | Health professionals | Lectures on rheumatology, medicine, diets, surgery, occupational and physical therapy, Arthritis Foundation information |
| Heuts, 2004 (54) | Hip or knee OA | 6-12 patients | 6 x 2 h  + booklet | Physical Therapists | Disease information, exercise, relaxation, communication, coping |

OA=osteoarthritis, NR= Not Reported

*Systematic literature search in Medline (PubMed, OVID), EMBASE and AMED databases for overviews and RCT’s on patient education / counselling / management and OA / osteoarthritis / osteoarthrosis / arthritis up until june 2011. Surgery related programmes were excluded. 110 abstracts were reviewed and 42 articles assessed in full-text.
